# Predicting 2-year time to progression in diffuse large B cell lymphoma using 3D CNNs on whole-body PET/CT scans

**DOI:** 10.1186/s13550-025-01336-1

**Published:** 2025-11-28

**Authors:** Maria C. Ferrández, Sanne E. Wiegers, Gerben J. C. Zwezerijnen, Martijn W. Heymans, Pieternella J. Lugtenburg, Jakoba J. Eertink, Lars Kurch, Andreas Hüttmann, Christine Hanoun, Ulrich Dührsen, Sally F. Barrington, N. George Mikhaeel, Luca Ceriani, Emanuele Zucca, Sándor Czibor, Tamás Györke, Martine E. D. Chamuleau, Josée M. Zijlstra, Ronald Boellaard, Sandeep S. V. Golla

**Affiliations:** 1 Cancer Center Amsterdam, Department of Radiology and Nuclear Medicine, Amsterdam UMC, Vrije Universiteit Amsterdam, Amsterdam, Netherlands; 2 Cancer Center Amsterdam, Imaging and Biomarkers, Amsterdam, Netherlands; 3 Cancer Center Amsterdam, Department of Hematology, Amsterdam UMC, Vrije Universiteit Amsterdam, Amsterdam, Netherlands; 4 Department of Epidemiology and Data Science, Amsterdam Public Health Research Institute, Amsterdam UMC, Vrije Universiteit Amsterdam, Amsterdam, The Netherlands; 5 Department of Hematology, Erasmus MC Cancer Institute, University Medical Center Rotterdam, Rotterdam, The Netherlands; 6 Department of Nuclear Medicine, Clinic and Polyclinic for Nuclear Medicine, University of Leipzig, Leipzig, Germany; 7 Department of Hematology, West German Cancer Center, University Hospital Essen, University of Duisburg-Essen, Essen, Germany; 8 School of Biomedical Engineering and Imaging Sciences, King’s College London and Guy’s and St Thomas’ PET Centre, King’s Health Partners, King’s College London, London, UK; 9 Department of Clinical Oncology, Guy’s Cancer Centre, School of Cancer and Pharmaceutical Sciences, King’s College London University, London, UK; 10 SAKK Swiss Group for Clinical Cancer Research, Bern, Switzerland; 11 Department of Nuclear Medicine, Medical Imaging Centre, Semmelweis University, Budapest, Hungary; 12 Amsterdam, The Netherlands

## Abstract

**Background:**

The aim of this study was to develop 3D convolutional neural networks (CNN) for the prediction of 2 years’ time to progression using PET/CT baseline scans from diffuse large B-cell lymphoma (DLBCL) patients. The predictive performance of the 3D CNNs was compared to that of the International Prognostic Index (IPI) and a previously developed 2D CNN model using maximum intensity projections (MIP-CNN).

**Results:**

1132 DLBCL patients were included from 7 independent clinical trials. Two 3D CNN models were developed using a training dataset of 636 patient scans merged from two trials, one CNN model trained on lesion-only PET (L-PET3D-CNN) and the second model trained on both lesion-only and whole body PET scans (LW-PET3D-CNN). The 3D models were cross-validated and performance was independently tested on 496 patient scans merged from five external trials, using the area under the curve (AUC). Performance was compared to the IPI and MIP-CNN using DeLong test. Occlusion maps were implemented to gain insights about the models’ decision-making process. The IPI and the MIP-CNN yielded an AUC of 0.53 and 0.65 respectively on external test data. The L-PET3D-CNN and the LW-PET3D-CNN yielded a significantly higher AUC, 0.65 and 0.64 respectively, compared to the IPI. For each individual external clinical trial, the models were consistently better than IPI. The MIP-CNN and the 3D CNNs showed equivalent performance on external test data.

**Conclusion:**

The 3D CNN models remained predictive of outcome on all external test datasets, outperforming the IPI. Although these models perform similarly to the MIP-CNN, the main advantage of the 3D CNN is the use of 3D occlusion maps to better understand the decision-making process of the models.

**Supplementary Information:**

The online version contains supplementary material available at 10.1186/s13550-025-01336-1.

## Introduction

In Medical Oncology, whole-body 2-[¹⁸F]fluoro-2-deoxy-D-glucose (^18^F-FDG) positron emission tomography combined with computed tomography (PET/CT) is the imaging technique of choice for diagnosing and monitoring treatment [1]. This is also the case in diffuse large B-cell lymphoma (DLBCL), where ^18^F-FDG PET/CT provides crucial anatomical and pathophysiological information associated with cancer progression and treatment outcome. Currently, one third of DLBCL patients will show tumor progression or relapse within the first 2 years after treatment [2]. In clinical practice, the early identification of high-risk patients mostly relies on the International Prognostic Index (IPI) and similar clinical scoring systems [3]. While the role of these scoring factors is limited [4], Artificial Intelligence (AI) and the use of convolutional neural networks (CNN) may offer the potential to uncover valuable insights that can aid in the prediction of tumor progression of DLBCL patients. One of the tasks at which deep learning models excel is automatic lesion segmentation and the computation of metabolic tumor volume (MTV) surrogates [5, 6]. These automated tools are of high interest since the extraction of PET parameters entails the delineation of the lesions, usually performed by a trained physician who needs to visually inspect the images slice-by-slice and delineate all the regions of interest manually. While automatic segmentation is becoming more sophisticated, it is possible to by-pass the segmentation step by developing deep learning models capable of predicting directly from the PET images. In our previous study, we developed a 2D CNN trained on maximum intensity projections (MIPs) of DLBCL PET scans which was predictive on 6 different external datasets from the PETRA database, on a total of 1132 patients [7, 8]. The use of MIPs were demonstrated to be a memory-efficient approach (vs. using whole scans) with promising results [9]. Using whole scans as inputs instead of MIPs requires the design of a 3D CNN, more computational power and longer training times [10]. With the growing use of deep learning models for prediction, their complexity is often not fully considered, making it more challenging to apply them effectively in clinical settings. Explainable AI (XAI) techniques can be used to provide greater transparency and insight into the model’s decision-making process. By making the inner layers of complex models more understandable, XAI helps users interpret, trust, and validate the results produced by AI systems. These techniques enable practitioners to identify potential biases, assess the reliability of the model, and ensure its fairness and accountability, thus providing greater confidence in AI applications across various domains [11–13].

In this study, two 3D CNN models which use PET scans as a single input to predict the probability of 2 years’ time to progression (TTP) of DLBCL patients are proposed. The models outcome is a binary prediction given by the probability of TTP longer than 2 years, P(TTP0), or TTP within 2 years, P(TTP1), where TTP1 indicates an increased risk of tumor progression for the patient. These models were trained using a total of 636 PET scans from two merged independent trials and externally tested on 5 independent clinical trials (496 images) from the PETRA database. The aim was to investigate the prognostic performance of such models and compare them to the IPI score (i.e. the current clinical standard) and to the MIP-CNN previously developed for the same task [7]. Furthermore, XAI was implemented to better understand the 3D CNN models’ outcomes using occlusion maps.

## Methods

### Datasets

Two datasets from the PETRA imaging database were used as training datasets: HOVON-84 [14] and PETAL [15]. The use of these datasets was approved by the institutional review board of the VU University Medical Center (JR/20140414). We found no significant differences in survival between the two IPI-corrected datasets [16].

*HOVON-84*. 373 ^18^F-FDG PET/CT baseline scans from DLBCL patients were included in the study (HOVON-84 trial: EudraCT, 2006–005,174 − 42). After inclusion/exclusion criteria [14], a total of 317 patients were available for this study. Missing DICOM information, failure to meet quality control (QC) requirements, incomplete scans or no FDG-avid lesions were the main reasons for patient exclusion. Patients who were lost to follow-up [7] or died of unrelated reasons within the first 2 years [14] were also excluded meaning 296 DLBCL patients were included.

*PETAL*. The PETAL trial provided 1098 ^18^F-FDG PET/CT baseline scans (PETAL trial: EudraCT 2006-001641−33) [15]. After exclusion (i.e. non-DLBCL patients, incomplete scans, missing DICOM information, QC out of range or no FDG-avid lesions) a total of 395 DLBCL scans were available for this study. Moreover, patients who had a different treatment to rituximab, cyclophosphamide, doxorubicin, vincristine and prednisone (R-CHOP) [12], were lost to follow-up within 2 years [24], and died of unrelated reasons within the first 2 years [19] were excluded, which led to 340 PETAL patients.

The 3D CNN models were externally validated using 5 completely independent trials, also from the PETRA database: GSTT15 [17], IAEA [18], NCRI [19], SAKK [20] and HOVON130 [21]. In total 847 ^18^F-FDG PET/CT baseline scans from newly diagnosed DLBCL patients were available from these 5 trials. After quality control, 496 scans were used in this study for external testing. Details on exclusion/inclusion criteria can be found in [8]. A description of patient characteristics for all clinical trials is given in Supplemental Table 1.

All patients were treated with R-CHOP with a varying number of cycles. Individual trials were approved by institutional review boards and all patients provided written informed consent. The institutional review board of the VU University Medical Center (JR/20140414) approved the use of this data.

*Quality Control of scans.* The participating sites provided the scans in DICOM format and these were subsequently anonymised. Mean standardized uptake value (SUVmean) of the liver was between 1.3 and 3.0 and, the plasma glucose level lower than 11 mmol/L as indicated in the QC criteria described by EANM guidelines [1]. The complete QC criteria followed is described in detail elsewhere [22].

## Three dimensional convolutional neural network

**Fig. 1:**
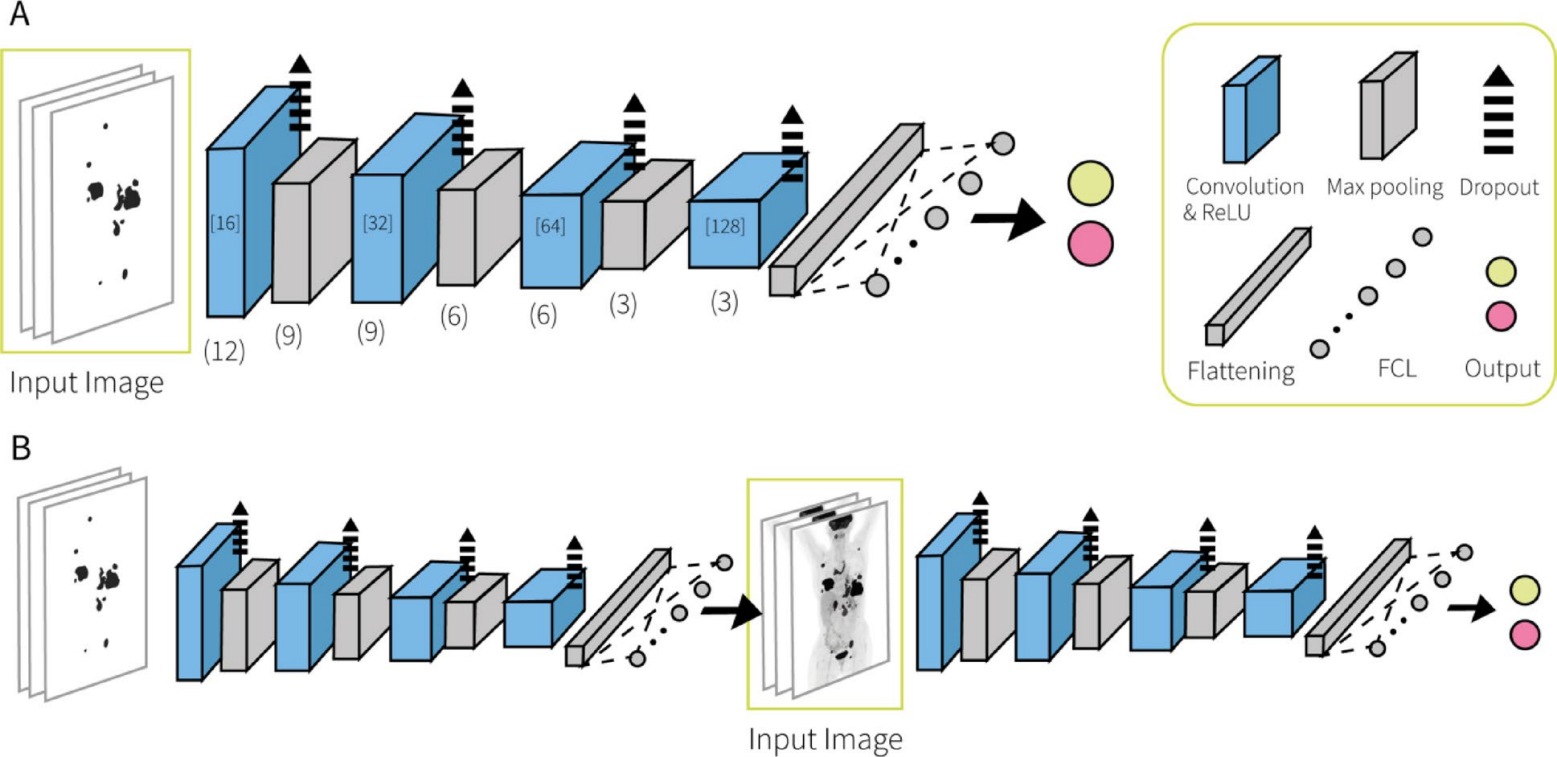
CNN architecture and training scheme. (**A**) Training scheme 1 (L-PET3D-CNN): the images containing only lesions are trained through 4 convolution layers. Lesion images are required for inference following training scheme 1. (**B**) Training scheme 2 (LW-PET3D-CNN): the images containing only lesions are trained through 4 convolution layers in the first step. In a second step, the model from the first step is further trained using the whole body 3D PET images using the same 3D CNN architecture of 4 convolution layers. Input images for L-PET3D-CNN model are the lesion-only images whereas for the LW-PET3D-CNN are the whole body images

*Architecture.* A four-layer convolutional design was used for the 3D CNN models. The number of feature maps starts at 16 in the first layer and increases up to 128 in the last layer, while their spatial dimensions progressively decrease by (3, 3, 3). Each convolutional layer uses the rectified linear unit (ReLU) activation function. To mitigate overfitting, a dropout rate of 35% was applied after each convolutional layer. This was followed by a MaxPooling layer, with pooling sizes of (3, 3, 3), (3, 3, 3) and (2, 2, 2) across the layers. Global Average Pooling was used in place of flattening, followed by a final fully connected layer. A softmax activation function was applied to generate class probabilities for the two output classes. The model was compiled using the Adam optimizer with a learning rate of 0.00005 and a decay rate of 0.000001. The overall 3D CNN architecture is illustrated in Fig. 1.

*Training.* In this study we trained the 3D CNN following two different training schemes. Training scheme 1 was defined by training on the PET images containing only lesion voxels (i.e. lesion-only PET) and is illustrated in Fig. 1A. Training scheme 2 was a two-step training where the model was first trained on the lesion-only PET images and followed by a second training step on the whole body PET images (Fig. 1B). In this scheme, the pre-trained weights from step 1 are used as initialization in step 2 further fine-tuning the model with the whole body PET images. These two schemes led to the development of two 3D CNN models, the Lesion-PET 3D CNN (L-PET3D-CNN, training scheme 1) and the Lesion-to-Whole PET 3D CNN (LW-PET3D-CNN, training scheme 2). The PET images used in training scheme 2 were previously processed to remove the brain region. This was done to provide consistency across the datasets since not all scans include the head segment. The main difference in their usage is that the L-PET3D-CNN uses the PET images containing only lesion information as inputs, whereas the LW-PET3D-CNN can predict directly from the whole body 3D PET scans.

In this study, 636 patients were used for model training. For the definition of TTP, patients who died within 2 years from the time of the baseline scan without signs of progression were excluded from the analysis.This led to 523 scans labeled as TTP0 from both HOVON84 (244) and PETAL (279) and only 113 scans labeled as TTP1 (HOVON84: 52, PETAL: 61). Class imbalance is a frequent problem in classification tasks within medical imaging research. We implemented a weighted cross entropy loss to address this issue [23]. In this case, the two different classes received a weight according to their frequency of appearance which was taken into account when updating the loss during backpropagation. The weighted cross entropy loss (WCELoss) is calculated as follows:

1 $$\:WCELoss\:=-\sum_{j=1}^{M}{w}_{j}{y}_{j}{\text{l}\text{o}\text{g}\left(p\right(y}_{j}\left)\right)$$

where *M* is the total number of classes and *y* is the label for class *j*. Taking *f(j)* as the frequency of class *j*, *w*_*j*_ is the weight assigned to class *j* and is calculated using Eq. (2).

2 $$\:{w}_{j}\:=\:\frac{{n}_{classes}}{{n}_{samples}\times\:f\left(j\right)}$$

The training data was divided into 2 sets on a patient level: training (80%) and test (20%). The test set remained as the hold-out set never seen by the model. The training set derived from the training data was again divided into training (80%) and validation (20%) sets in order to perform a 5 fold cross validation (CV) scheme to provide a reliable estimate of the models performance and to reduce the variance in the reported metrics. In training scheme 1, the model was trained on the Lesion PET images for 1200 epochs. In training scheme 2, the model was trained in two steps with 1200 epochs each time. The batch size was set to 14. This was the maximum capacity possible with our GPU system. The model training and evaluation were conducted on an NVIDIA HGX system equipped with 8 × NVIDIA A100-SXM4-80GB GPUs. All models were implemented using Python version 3.9.16, Keras version 2.10.0 and Tensorflow library version 2.10.0.

## Pre processing

All PET scans were standardized to a size of 275 × 200 × 200 and a voxel size of 4 × 4 × 4 mm. The scans were normalized by a fixed maximum intensity value (SUV = 40). The values above this maximum were truncated to avoid normalization to be driven by the SUV value of high uptake organs (i.e. bladder).

To generate the lesion images (scans which only contain lymphoma lesion(s) segmentation) the ACCURATE tool was used [24]. The segmentation of the tumours was performed using a SUV threshold of 4.0 and any physiological uptake adjacent to tumours was manually deleted [22]. SUV4.0 is the benchmark segmentation method for DLBCL indicated in [25]. All scans were reviewed by a nuclear medicine physician, and delineations were performed under supervision of a nuclear medicine physician.

Brain removal was performed using a region growing algorithm. A seed was automatically placed in the hottest voxel along the centre of the x and the z axes (x, z = 100) as an estimation of the brain location. Voxels placed in 6 × 6 neighbourhood around the seed and across the 3 axes were appended based on a standard uptake value threshold. The algorithm explored several thresholds (SUV of 4, 2.5 and 2) and selected the optimal one based on the size of the selected region. A mask was generated out of the grown region. The mask was dilated and used to remove the brain from the scans.

Lesions near the brain region can occur in DLBCL patients. These can easily be missed or truncated when removing the brain. To avoid this, mask lesions were generated by thresholding the scans at different SUVs. These masks were then used to recover any partially truncated lesions during the process of removing the brain.

### Statistical analysis

The receiver-operator characteristic (ROC) curve and the area under the curve (AUC) were used to evaluate the 3D CNN models’ performance. The average AUC and standard deviation (SD) across the five folds was reported as the estimated performance of the 3D CNN models for all three sets derived from the training dataset: training, validation and testing. Sensitivity and specificity were calculated across the 5 folds using the Youden index as a threshold. 95% confidence intervals (CIs) for the AUC were obtained using Monte Carlo stratified subsampling (1000 iterations), where 80% of samples were randomly drawn without replacement in each iteration. The best performing model from the 5-fold CV was considered the final model and used for external validation on the 5 datasets. AUC for each external dataset was reported as well as the performance in the merged datasets. Two-sided Delong test was used to compare the AUC obtained with L-PET3D-CNN and LW-PET3D-CNN models on all 5 external datasets to that of the IPI and the MIP-CNN model [26].

## Explainable AI: occlusion sensitivity

Occlusion Sensitivity is a perturbation-based method where different regions of an image are patched or occluded to assess the impact that these may have on the final prediction [11]. A sensitivity score is computed for each occluded region which accounts for the change in the classification score. The variation in the sensitivity scores can be used to generate a heatmap highlighting the areas of the image where occlusion causes the largest variations in the models prediction. The dimensions of the patch (i.e. occluded region) depend on multiple factors such as image size or regions of interest. In this study we used a patch of size (5, 5, 5) with a stride of 5 and the patched region was replaced by 0s. The heatmap is then the result of all the sensitivity scores arranged by region and the sensitivity score was calculated as the change between the original predicted value and the new predicted value after occlusion. A higher sensitivity score means that the occluded region contributed significantly to the classification score (i.e. final prediction) and a lower sensitivity score means that the occluded region has a lower contribution or no contribution at all.

## Results

### Prediction models

**Fig. 2:**
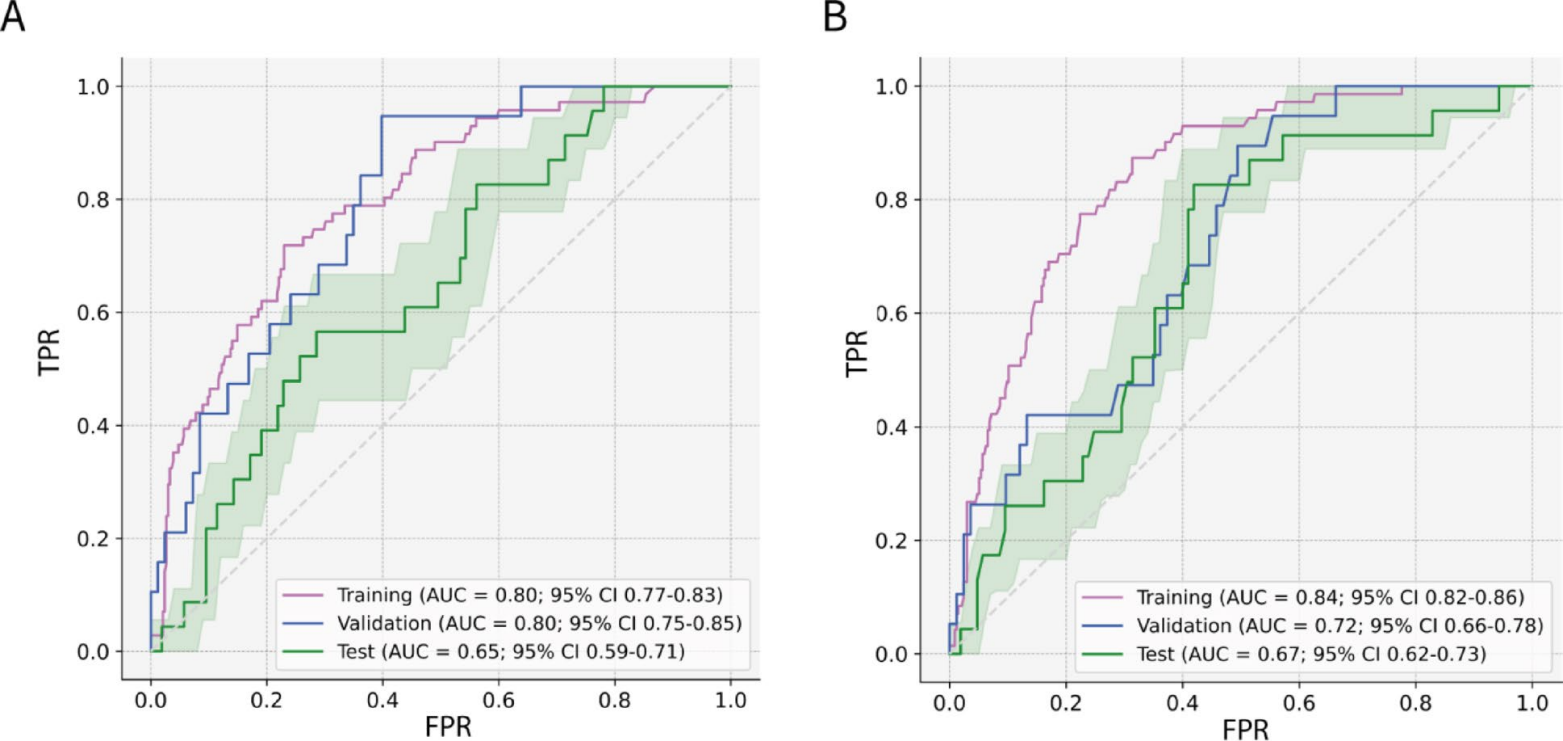
ROC and AUC for training, validation and test set. (**A**) L-PET3D-CNN model. (**B**) LW-PET3D-CNN model. The green shaded area represents the 95% confidence interval for the test ROC curve. 95% confidence intervals were estimated using Monte Carlo stratified subsampling

The results for the 5-fold CV for the L-PET3D-CNN and the LW-PET3D-CNN are shown in Table 1, Supplemental Tables 2 and 3. The standard deviation, sensitivity and specificity values are also reported in Table 1. The model derived from fold 0 resulted in the best performing model on average for both the L-PET3D-CNN and the LW-PET3D-CNN (Fig. 2).

**Fig. 3:**
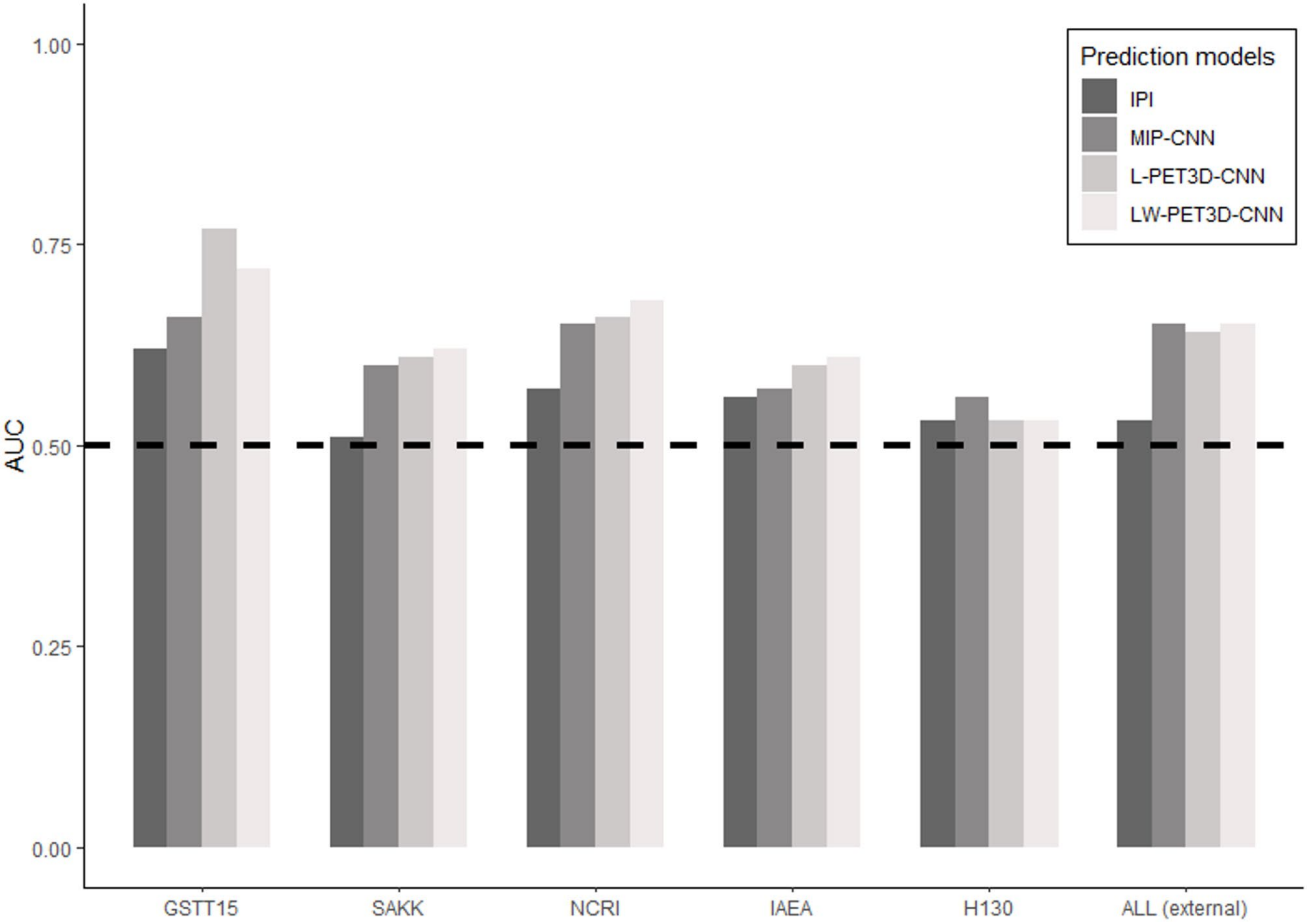
AUC values of IPI, MIP-CNN, L-PET3D-CNN and LW-PET3D-CNN prediction models for all 5 external datasets (*n* = 496 external validation dataset)

The individual AUCs of each separate external dataset are shown in Fig. 3 (and Supplemental Table 4) together with the AUC yielded by all 496 patients from the 5 merged external datasets compared to the IPI and the MIP-CNN that was developed in an early study [7]. The external validation on 496 patients yielded an AUC of 0.53 for the IPI model whereas the MIP-CNN achieved an AUC of 0.65, which was significantly higher than the IPI model (*P* < 0.03). The L-PET3D-CNN and the LW-PET3D-CNN achieved an AUC of 0.64 and 0.65 respectively, also significantly higher than that of the IPI (*P* < 0.03). No statistical differences were found between the MIP-CNN and the two models explored in this study. From all 5 external studies, we only found a significantly improved performance for GSTT15 with the L-PET3D-CNN and the LW-PET3D-CNN compared to the MIP-CNN (*P* < 0.04 and *P* < 0.005, respectively). The performance of L-PET3D-CNN and LW-PET3D-CNN was statistically equivalent in all 5 datasets.

**Table 1:** Cross validated AUC, standard deviation, sensitivity and specificity values for the L-PET3D-CNN and LW-3DPET-CNN for training, validation and test set (*n* = 636 training dataset)

Model	Set	AUC (SD)	Sensitivity* (SD)	Specificity* (SD)	95% CI**
L-PET3D-CNN	Training	0.80 (0.01)	0.77 (0.10)	0.70 (0.09)	[0.78–0.81]
	Validation	0.78 (0.08)	0.72 (0.18)	0.68 (0.12)	[0.74–0.80]
	Test	0.63 (0.02)	0.58 (0.06)	0.64 (0.09)	[0.60–0.65]
LW-PET3D-CNN	Training	0.75 (0.07)	0.80 (0.07)	0.61 (0.15)	[0.74–0.76]
	Validation	0.67 (0.06)	0.69 (0.08)	0.58 (0.12)	[0.61–0.68]
	Test	0.64 (0.03)	0.67 (0.12)	0.55 (0.11)	[0.61–0.66]

*Values based on Youden index

** Estimated using Monte Carlo stratified subsampling

SD: standard deviation, CI: confidence intervals

## XAI: Occlusion maps

**Fig. 4:**
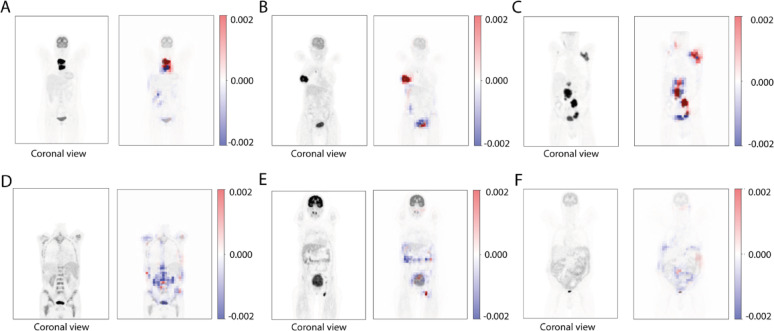
Coronal views of six different patients and their corresponding occlusion map heatmaps for the LW-PET3D-CNN model. (**A**–**C**) represent three patients with clearly localized tumors, categorized as straightforward cases. (**D**–**F**) depict three patients with no distinct tumor masses, classified as complex cases. In (**D**), the tumor is infiltrating the bone. In (**E**), a full enlarged bladder is observed. In (**F**), the tumor shows extensive bowel/peritoneal infiltration with ill-defined borders. Regions in red have the strongest contribution to the model’s prediction

Figure 4 shows six examples of the occlusion maps output for the LW-PET3D-CNN. Regions in bright red have the strongest contribution to the model’s prediction. Conversely, regions in blue may represent a negative impact to the models prediction. Generally, we see that blue regions are scarce and in a lighter shade compared to the red regions. Red regions are always located within or nearby tumor locations. Occlusion maps for model L-PET3D-CNN are shown in Supplemental Fig. 1

## Discussion

In this study, two 3D CNN models for the prediction of time to progression in DLBCL PET images were developed. Generalizability of these models was evaluated on 5 independent clinical trials from the PETRA database. We found that both the 3D CNN models were predictive of outcome and outperformed IPI for all training and external datasets.

In our previous study, we developed a 2D CNN trained with MIPs instead of the whole PET scans, which also outperformed IPI [8]. The use of MIPs is an efficient resource to cope with the computational expense of deep learning models and medical imaging however, information-loss can occur. This study was intended to address this issue with the design and development of the 3D CNN model(s). The L-PET3D-CNN and the LW-PET3D-CNN performance remained similar to that of the MIP-CNN in most of the datasets. We only found a significant improvement performance for GSTT15 (MIP-CNN: 0.66, L-PET3D-CNN: 0.72, LW-PET3D-CNN: 0.77). No significant difference was observed in the rest of the external datasets. It is difficult to pinpoint a specific reason for this outcome. However, we hypothesize that dataset specific characteristics may play a role. The GSTT15 dataset may include imaging or clinical features (e.g., lesion distribution, background activity, scanner-specific reconstruction differences) that favor the model that utilizes full 3D context. Moreover, the average performance of the 3D CNN models and the 2D MIP-CNN remained statistically equivalent on all 496 external scans. These findings suggest that information-loss related to MIPs may not be as concerning as initially expected in the case of DLBCL patients.

The use of deep learning for the classification and segmentation of PET images in the medical oncology field has increased drastically in the past few years. For DLBCL, most AI-based methods focus on tumor segmentation while only a few studies have explored the use of deep learning models for outcome prediction [5, 9, 10, 27]. The 3D CNN developed by Liu et al. trained on DLBCL FDG-PET scans yielded a promising cross-validated training performance (AUC = 0.82), comparable to our results. However, no external testing performance was presented. For both the L-PET3D-CNN and LW-PET3D-CNN models, the performance on the test data is lower than that of the training/validation data but overall consistent with that of the MIP-CNN and other radiomic models [8]. There are case-mix differences in the data across clinical trials which seem to consistently impact the performance of all models. Specific patient characteristics not considered during model development could be the reason for these differences. These characteristics were thoroughly discussed in our previous study [8].

Herein we have described two different training schemes for the development of a 3D CNN for the outcome prediction of DLBCL patients. Both led to comparable results in terms of performance. However, the L-PET3D-CNN uses the PET images containing only lesion information as inputs, whereas the LW-PET3D-CNN can predict directly from the whole body 3D PET scans. This means that for the implementation of the L-PET3D-CNN, segmentation of the PET images is required. SUV4.0 segmentation is the benchmark in DLBCL lesion segmentation [25]. However, this method requires user input as low-uptake and/or smaller lesions are added manually and therefore, it may introduce bias.

Even though the 2D MIP-CNN might be a smart choice when predicting outcome in DLBCL, the 3D models developed in this study have a clear advantage over our 2D MIP-CNN. The architecture of the 3D CNN is different to that of the 2D MIP-CNN as the latter consists of two branches to take both coronal and sagittal MIPs but the 3D CNN only needs one single branch as shown in Fig. 1. The 3D CNN design facilitates the implementation of XAI techniques such as occlusion maps which help to understand the model’s predictions. We selected occlusion sensitivity analysis as our XAI approach because it offers an intuitive and practical way to highlight image regions most relevant for predictions. We also explored Grad-CAM, but due to the downsampling layers of our CNN, the resulting feature maps had very low spatial resolution and were less interpretable. By contrast, occlusion analysis provided higher-resolution and clinically plausible maps (Fig. 4). We analyzed several PET images and found that generally tumor regions had the largest impact on the prediction values (red regions). The occlusion of these regions led to a decrease in the prediction values (i.e. lower risk of tumor progression). Moreover, this impact is not uniform across the lesions, certain regions within the lesions seem to have greater impact than others denoted by the different intensities of red. There are also certain areas which showed a negative impact (blue regions), meaning that the occlusion of such regions resulted in a higher prediction value (i.e. higher risk of progression). The blue areas are usually located in the healthy tissue surrounding the tumors and within/around the bladder (Fig. 4B and C). In some cases, the blue regions appear scattered across different healthy organs (Fig. 4D and E). When the tumor is largely disseminated with no clear tumor boundaries, the red regions become lighter probably because the model finds it more difficult to identify the relevant regions (Figs. 4D and F). A similar pattern is observed in Supplemental Fig. 1. In this case, the highlighted regions are fewer and lighter. The interpretation of the blue regions is a complex problem as little is known about the biological effect of healthy tissue, organs and the surroundings of the tumor on the progression of the tumor. Nonetheless, the generation of such images using occlusion maps may give insight into the decision-making process of the model which is often a challenge.

There are some limitations in this study that need to be addressed. The training of these 3D models is computationally expensive and time consuming and due to the size of the PET images, large GPU memory footprint was required. The majority of patients in this study received standard R-CHOP treatment; however, some variations in treatment regimens were noted across studies, including differences in the number of cycles and the level of treatment intensification. As the data included in this study are retrospective, some residual inter-center differences may remain, which could have influenced the models’ performance.

Overall, this is the first study to show the potential of 3D CNNs for predicting the probability of 2-year TTP and their application in an extensive cohort of ^18^F-FDG PET baseline DLBCL patient scans whilst providing some degree of clarity on the model’s inferential process. The model was developed by extensive training and cross-validation and tested in new external data, which is a strength.

## Conclusion

In this study we developed two 3D CNN models, the L-PET3D-CNN and LW-PET3D-CNN, capable of predicting the probability of 2-year TTP in DLBCL patients using ^18^F-FDG PET/CT scans as input. Both models remained predictive of outcome in 5 independent external datasets, outperforming the IPI scores. These models perform similarly to the 2D MIP-CNN, a model that uses MIPs as inputs. The main advantage of the 3D CNN design is that it enabled the use of an explainable AI method to better understand the predictions provided by the models.

## Supplementary Information

Supplementary Material 1

Supplementary Material 2

Supplementary Material 3

Supplementary Material 4

Supplementary Material 5

Supplementary Material 6

## Data Availability

The datasets generated during and/or analyzed during the current study are available from the corresponding author on reasonable request.
