# Supplementary material for: Predicting 2-year time to progression in diffuse large B cell lymphoma using 3D CNNs on whole-body PET/CT scans
Source: EJNMMI Res. 2025 Nov 28;15:140. doi: 10.1186/s13550-025-01336-1 (PMC12662970; doi:10.1186/s13550-025-01336-1)
Supplement: Supplementary file 1 — Supplementary Material 1 [file 13550_2025_1336_MOESM1_ESM.docx]

**
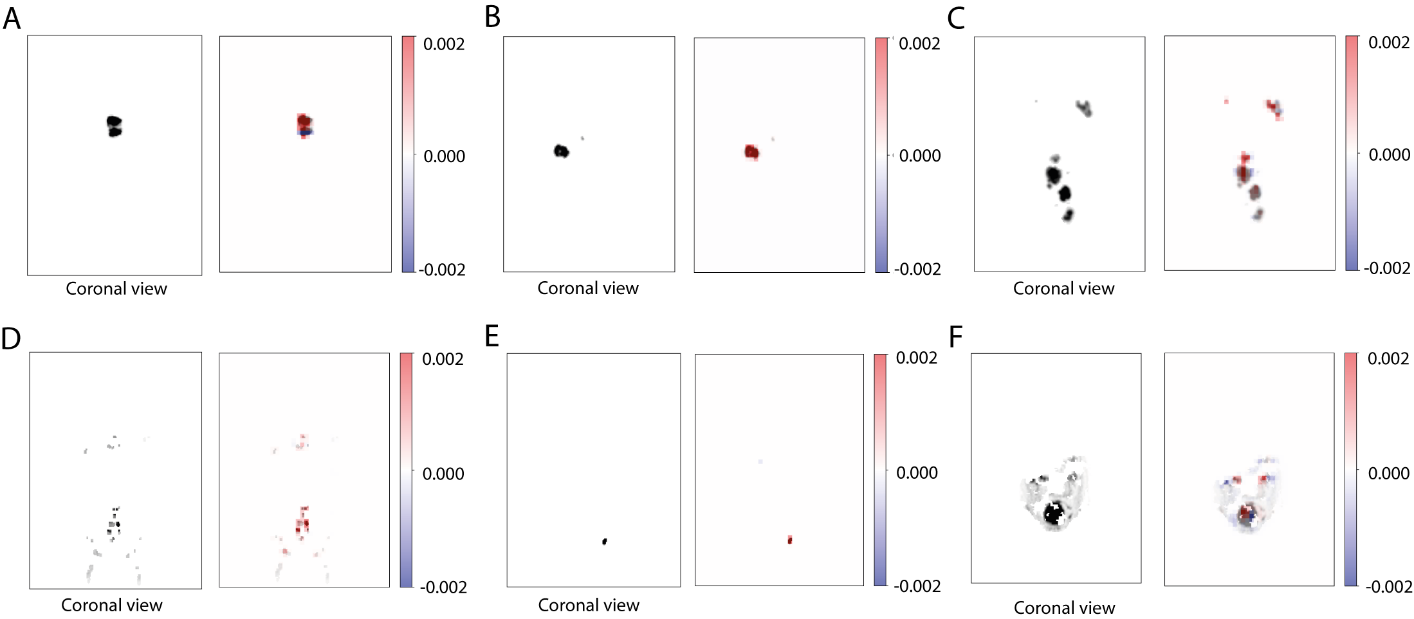
**

**Supplemental Figure 1**. Coronal views of six different patient’s lesion-only scans and their corresponding occlusion map heatmaps for the L-PET3D-CNN model. (A–C) represent three patients with clearly localized tumors, categorized as straightforward cases. (D–F) depict three patients with no distinct tumor masses, classified as complex cases. In (D), the tumor is infiltrating the bone. In (E), a full enlarged bladder is observed. In (F), the tumor shows extensive bowel/peritoneal infiltration with ill-defined borders**.** Regions in red have the strongest contribution to the model’s prediction.
