# Supplementary material for: Predicting 2-year time to progression in diffuse large B cell lymphoma using 3D CNNs on whole-body PET/CT scans
Source: EJNMMI Res. 2025 Nov 28;15:140. doi: 10.1186/s13550-025-01336-1 (PMC12662970; doi:10.1186/s13550-025-01336-1)
Supplement: Supplementary file 2 — Supplementary Material 2 [file 13550_2025_1336_MOESM2_ESM.docx]

**Supplemental Table 1**. Patient characteristics per clinical trial.

|  | **H84**  **(N = 296)** | **PETAL (N = 340)** | **GSTT15**  **(N = 95)** | **SAKK**  **(N = 109)** | **NCRI**  **(N = 123)** | **IAEA**  **(N = 104)** | **H130**  **(N = 65)** |
| --- | --- | --- | --- | --- | --- | --- | --- |
| **Characteristic** | **N (%)** | **N (%)** | **N (%)** | **N (%)** | **N (%)** | **N (%)** | **N (%)** |
| **Age**  Median (IQR)  ≤ 60 years  > 60 years | 65 (55-72)  97 (33)  199 (67) | 60 (50-69)  172 (50)  168 (49) | 61 (49-70)  46 (48)  49 (52) | 59 (48-67)  59 (54)  50 (46) | 61 (48-68)  60 (49)  63 (51) | 56 (43-65)  64 (61)  42 (39) | 63 (54-72)  30 (46)  35 (54) |
| **Sex** Male  Female | 152 (51)  144 (49) | 194 (57)  146 (43) | 45 (47)  50 (53) | 57 (52)  52 (48) | 74 (60)  49 (40) | 55 (54)  49 (47) | 37 (57)  28 (43) |
| **Ann Arbor stage**  I  II  III  IV  missing | 0 (0)  48 (16)  62 (21)  186 (63) | 65 (19)  79 (23)  65 (19)  130 (38)  1 (1) | 9 (9)  20 (21)  11 (12)  55 (58) | 14 (13)  39 (36)  25 (23)  31 (28) | 8 (6)  49 (40)  30 (24)  36 (30) | 11 (11)  25 (24)  23 (22)  45 (43) | 0  7 (11)  8 (12)  50 (77) |
| **LDH** normal  > normal  missing | 97 (33)  199 (67) | 148 (44)  191 (55)  1 (1) | 35 (37)  60 (63) | 59 (52)  55 (48) | 48 (39)  75 (61) | 54 (52)  50 (48) | 16 (25)  45 (69)  4 (6) |
| **Extranodal localizations** ≤1 >1  missing | 175 (59)  121 (41) | 239 (70)  99 (29)  2 (1) | 45 (47)  50 (53) | 89 (77)  25 (23) | 100 (81)  23 (19) | 67 (64)  37 (36) | 30 (46)  35 (54) |
| **WHO**  **Perf. status**  0  1  2  3  4 | 172 (58)  87 (29)  37 (13)  0 (0)  0 | 156 (46)  150 (44)  25 (7)  9 (3)  0 | 32 (33)  35 (37)  16 (17)  12 (13)  0 | 65 (60)  37 (34)  7 (6)  0  0 | 72 (58)  38 (31)  13 (11)  0  0 | 36 (35)  44 (42)  15 (14)  7 (7)  2 (2) | 38 (58)  23 (35)  3 (5)  1 (2)  0 |
| **IPI** Low  Low-intermediate  High-intermediate  High | 48 (16)  73 (25)  103 (35)  72 (24) | 123 (36)  92 (27)  84 (25)  40 (12) | 26 (27)  10 (11)  29 (30)  30 (32) | 57 (52)  21 (20)  17 (15)  14 (13) | 51 (41)  23 (19)  32 (26)  17 (14) | 44 (43)  16 (15)  22 (21)  22( 21) | 9 (14)  14 (22)  29 (45)  13 (20) |
| **R-CHOP cycles**  2 to 5  6  7  8 | 10 (3)  123 (40)  2 (1)  161 (56) | 19 (6)  300 (88)  3 (1)  18 (5) | 18 (19)  74 (78)  0  3 (3) | 4 (4)  105 (96)  0  0 | 8 (6)  12 (11)  0  103 (83) | 13 (12)  43 (41)  4 (5)  44 (42) | unknown  unknown  unknown  unknown |

*Abbreviations. IQR: Interquartile Range, LDH: serum lactate dehydrogenase level, WHO: World Health Organization, IPI: International Prognostic Index, R-CHOP: rituximab, cyclophosphamide, doxorubicin, vincristine and prednisone*
