# Supplementary material for: Predicting 2-year time to progression in diffuse large B cell lymphoma using 3D CNNs on whole-body PET/CT scans
Source: EJNMMI Res. 2025 Nov 28;15:140. doi: 10.1186/s13550-025-01336-1 (PMC12662970; doi:10.1186/s13550-025-01336-1)
Supplement: Supplementary file 3 — Supplementary Material 3 [file 13550_2025_1336_MOESM3_ESM.docx]

**Supplemental Table 2.** AUC, standard deviation, sensitivity and specificity values for the LW-PET3D-CNN for training, validation and test set.

|  | Training | | | Validation | | | Test | | |
| --- | --- | --- | --- | --- | --- | --- | --- | --- | --- |
|  | AUC | Sensitivity | Specificity | AUC | Sensitivity | Specificity | AUC | Sensitivity | Specificity |
| Fold 0 | 0.84 | 0.86 | 0.69 | 0.72 | 0.63 | 0.60 | 0.67 | 0.65 | 0.59 |
| Fold 1 | 0.77 | 0.81 | 0.63 | 0.67 | 0.61 | 0.68 | 0.65 | 0.65 | 0.60 |
| Fold 2 | 0.74 | 0.76 | 0.63 | 0.74 | 0.81 | 0.59 | 0.64 | 0.61 | 0.56 |
| Fold 3 | 0.65 | 0.86 | 0.35 | 0.60 | 0.71 | 0.38 | 0.60 | 0.87 | 0.36 |
| Fold 4 | 0.77 | 0.71 | 0.76 | 0.62 | 0.67 | 0.63 | 0.63 | 0.56 | 0.65 |
