# Supplementary material for: Predicting 2-year time to progression in diffuse large B cell lymphoma using 3D CNNs on whole-body PET/CT scans
Source: EJNMMI Res. 2025 Nov 28;15:140. doi: 10.1186/s13550-025-01336-1 (PMC12662970; doi:10.1186/s13550-025-01336-1)
Supplement: Supplementary file 4 — Supplementary Material 4 [file 13550_2025_1336_MOESM4_ESM.docx]

**Supplemental Table 3.** AUC, standard deviation, sensitivity and specificity values for the L-PET3D-CNN for training, validation and test set.

|  | Training | | | Validation | | | Test | | |
| --- | --- | --- | --- | --- | --- | --- | --- | --- | --- |
|  | AUC | Sensitivity | Specificity | AUC | Sensitivity | Specificity | AUC | Sensitivity | Specificity |
| Fold 0 | 0.80 | 0.69 | 0.77 | 0.80 | 0.58 | 0.78 | 0.65 | 0.56 | 0.70 |
| Fold 1 | 0.79 | 0.89 | 0.59 | 0.80 | 0.87 | 0.53 | 0.61 | 0.65 | 0.54 |
| Fold 2 | 0.79 | 0.68 | 0.76 | 0.82 | 0.81 | 0.77 | 0.65 | 0.52 | 0.71 |
| Fold 3 | 0.79 | 0.88 | 0.59 | 0.83 | 0.88 | 0.57 | 0.63 | 0.65 | 0.54 |
| Fold 4 | 0.82 | 0.73 | 0.77 | 0.64 | 0.47 | 0.76 | 0.62 | 0.52 | 0.71 |
