# Supplementary material for: Predicting 2-year time to progression in diffuse large B cell lymphoma using 3D CNNs on whole-body PET/CT scans
Source: EJNMMI Res. 2025 Nov 28;15:140. doi: 10.1186/s13550-025-01336-1 (PMC12662970; doi:10.1186/s13550-025-01336-1)
Supplement: Supplementary file 5 — Supplementary Material 5 [file 13550_2025_1336_MOESM5_ESM.docx]

**Supplemental Table 4**. AUC values of IPI, MIP-CNN, L-PET3D-CNN and LW-PET3D-CNN prediction models for all 5 external datasets (n=496 external validation dataset).

| **DATASET** | **IPI** | **2D MIP-CNN** | **L-PET3D-CNN** | **LW-PET3D-CNN** |
| --- | --- | --- | --- | --- |
| IAEA | 0.56 | 0.57 | 0.60 | 0.61 |
| SAKK | 0.51 | 0.60 | 0.61 | 0.62 |
| GSTT15 | 0.62 | 0.66 | 0.77 | 0.72 |
| NCRI | 0.59 | 0.65 | 0.66 | 0.68 |
| H130 | 0.53 | 0.56 | 0.53 | 0.53 |
| ALL (5 external datasets) | 0.53 | 0.65 | 0.64 | 0.65 |
