# Supplementary material for: Predicting 2-year time to progression in diffuse large B cell lymphoma using 3D CNNs on whole-body PET/CT scans
Source: EJNMMI Res. 2025 Nov 28;15:140. doi: 10.1186/s13550-025-01336-1 (PMC12662970; doi:10.1186/s13550-025-01336-1)
Supplement: Supplementary file 6 — Supplementary Material 6 [file 13550_2025_1336_MOESM6_ESM.docx]

**Submission ID#: EJRE-D-25-00295**

**Title: Predicting 2-Year Time to Progression in diffuse large B cell lymphoma Using 3D CNNs on Whole-Body PET/CT Scans.**

The authors would like to thank the reviewers for their valuable time and constructive comments which have helped to improve the quality of the manuscript. A more detailed response is indicated below following the specific reviewer’s comments. The text inserted in the manuscript is indicated in italics. All changes in the manuscript are listed in red.

*Reviewer #1: I have read with interest the manuscript by Ferrandez et al. that investigates the predictive performance of two 3D convolutional neural networks (CNNs) to predict 2 years' time to progression using FDG-PET/CT baseline scans for diffuse large B-cell lymphoma (DLBCL) patients. The performance of these 3D CNNs (one that uses information from just the tumour and one that uses the whole-body PET scans) was compared to a previously developed 2D CNN using maximum intensity projections (MIP-CNN) and a clinical International Prognostic Index (IPI) model. The models were trained on a large dataset (636 patients) and validated on a large external dataset (496 patients). The authors show that the 3D CNNs perform similarly to the 2D CNN, but all CNNs perform better than the IPI model. Although the 3D CNNs do not outperform the previously published 2D CNN, the authors show that the 3D CNN offers opportunities to use explainable AI (XAI) to illustrate what triggers the CNN to make certain decisions.*

*Major comments:*

*1) AUCs of 0.80 and higher are considered clinically useful and acceptable. When these are below, the methods are considered of limited clinical utility. The CNNs have an AUC of 0.64-0.65. How useful would these techniques be in clinical practice, considering that these are well below 0.80? Please discuss.*

We thank the reviewer for raising this important point. Our CNN models achieved AUCs in the range of 0.64–0.65, which indeed fall below this threshold. However, it is important to note that these values are higher than the performance of the International Prognostic Index (IPI), which remains the most widely used tool in clinical practice and typically achieves AUC values between 0.50-0.60.This suggests that, although not reaching an AUC of 0.80, our CNN-based approach already surpasses the prognostic accuracy of the standard-of-care clinical index.

Importantly, this study was designed as a proof-of-concept to evaluate whether CNNs can extract prognostic information from whole body PET scans. While the observed performance is modest, these results are still informative in the sense that they demonstrate that CNNs can extract predictive information from the PET scans and provide a baseline for further model development and optimization which may improve performance in future studies. We believe that, even though these models are not yet suitable for clinical decision-making, this study highlights specific challenges (e.g. dataset size, heterogeneity, model interpretability) that might need to be addressed before reaching clinically acceptable AUC values.

*2) There are many XAI methods available that may be useful for nuclear medicine scans (De Vries et al., Front Med, 2023). Why was occlusion sensitivity chosen in this study as the XAI method? Are the results from Figure 4 reliable for interpretation? And why can't this method (or another XAI method) be used for MIP-CNN? Please discuss.*

We thank the reviewer for bringing up this topic regarding model explanability. We are aware that many approaches have been proposed for medical imaging applications (as reviewed by De Vries et al., 2023). Among these, we selected occlusion sensitivity analysis because it provides a direct way to assess the influence of localized image regions on the models predictions. Moreover, it is an established method in medical imaging and the output is relatively straightforward to interpret.

We also explored Grad-CAM as an alternative. However, because of the architecture of our CNN (specifically the downsampling layers), Grad-CAM produced very low spatial resolution outputs which made them difficult to interpret. By contrast, occlusion maps provided more detailed and clinically plausible maps. We have indicated this in the Discussion section to clarify this methodological choice.

Regarding Figure 4, we agree that no XAI method offers a complete understanding of CNN decision-making. Nevertheless, the occlusion maps shown were mostly consistent across patients and highlighted regions consistent with clinical expectations, making them reliable for qualitative interpretation within the limitations of this method.

As the reviewer notes, we did not apply occlusion sensitivity to the **MIP-CNN**. This was primarily due to its two-branch design (sagittal and coronal inputs) that are concatenated later in the network, which complicates pixel-level perturbation analysis. While it is theoretically possible to adapt occlusion or apply other approaches, this would require substantial architectural adjustments and was beyond the scope of the study.

*3) Only significantly improved performance for 3D CNNs compared to MIP-CNN for GSTT15 was observed. Could the authors find a reason for this? Did the occlusion maps provide an indication of why 3D CNN would likely provide better results than MIP-CNN compared to other studies? Please discuss.*

We thank the reviewer for this valuable comment. Indeed, the significantly improved performance of the 3D CNN compared to the MIP-CNN only occurred for the case of the GSTT15 dataset. It is difficult to pinpoint a specific reason for this outcome however we hypothesize that dataset specific characteristics may play a role. The GSTT15 dataset may include imaging or clinical features (e.g., lesion distribution, background activity, scanner-specific reconstruction differences) that favor the model that utilizes full 3D context. We have included this in the Discussion section.

The occlusion sensitivity analysis (Figure 4) consistently highlighted regions corresponding to metabolically active tumor sites, suggesting that the 3D CNN effectively captured lesion-related signals. It could be that for this particular dataset the MIP-CNN may have struggled to localize individual lesions once compressed into 2D projections.

We acknowledge, however, that these explanations remain speculative and that the observed difference could also be influenced by other factors.

*4) Several studies are included. How were the data harmonized between centers, scanners and/or datasets? If harmonization was not applied, could this have influenced your results? Please discuss.*

We thank the reviewer for raising this important point. We did apply several harmonization steps across datasets to reduce variability between centers and scanners. Specifically, we (i) resampled all PET images to a common voxel size and spatial dimension, and (ii) normalized the standardized uptake values (SUVs) to a fixed range, truncating values above 40 to minimize the influence of extreme outliers. These steps ensured that the CNNs received input data in a uniform format across all cohorts.

We agree that some residual inter-center differences (e.g., related to patient preparation, acquisition protocols, or scanner generations) may remain, and these could have influenced model performance. We have added this as a limitation to the Discussion section.

*Minor comments:*

*1) Abstract, lines 44-49: The conclusion is fair, but with some minor rewriting it would be easier to read. Suggestion: "Although these models perform similarly to the MIP-CNN, the main advantage of the 3D CNN is the use…"*

We want to thank the reviewer for the suggestion. We have modified the abstract accordingly.

*2) Introduction, line 2, "whole-body 18F-fluorodexoyglucose": Please follow the EANM tracer nomenclature ("Let's be precise: A practical short guidance to correct radiotracer nomenclature in nuclear medicine," EANM, January 2024), and use either: 2-deoxy-2-[18F]fluoro-D-glucose or 2-[18F]fluoro-2-deoxy-D-glucose.*

We thank the reviewer for pointing this out. This has been corrected in the introduction.

*3) Methods, pre-processing, line 1, "was manually deleted": Who performed the manual deletions? What was the level and/or years of experience of this person? If the person was not highly experienced, who supervised or verified the manual operations?*

The manual deletions were all reviewed and supervised by a nuclear medicine physician. This has been added to the methods section of the manuscript.

*4) References, refs 9, 11, 12, 20: Please provide complete details for these references and/or use the correct abbreviations for the journal names.*

We thank the reviewer for pointing this out. We have made the corresponding changes in the references.

*Reviewer #2: This work aims to report the development of 2 deep learning networks (CNNs) with predictive performance in terms of 2 years PFS in cohorts of DLBCL patients using imaging PET. The aim of this paper is clear enough. The methods are well described, and results seem well-reported.*

*1) I suggest adding the Confidence intervals to all AUC curves calculated, especially in fig. 2.*

We thank the reviewer for this helpful suggestion. In the revised manuscript, we have added 95% confidence intervals (CIs) to the ROC curves shown in **Figure 2**. In addition, we now report 95% CIs for the AUC values in the **training performance table (Table 1)**. The CIs were estimated using Monte Carlo stratified subsampling (1000 iterations) as noted in Methods section, figure description and table footnote.

*2) Why did the authors choose the occlusion maps technique? I mean, this is not a methodological error, but Grad-CAM maps have been used in publications employing PET imaging. A methodological discussion about this choice could help.*

We thank the reviewer for raising this point. As noted in our response to Reviewer 1, we explored different explainable AI methods for our CNN models. While Grad-CAM has indeed been applied in PET imaging, in our case the resulting maps had very low spatial resolution due to the architecture of our network (in particular, the downsampling layers), which made them less interpretable. By contrast, occlusion sensitivity provided higher-resolution and clinically more plausible maps, which is why we selected this method for presentation. We have indicated this in the Discussion section to clarify this methodological choice.

*3) The following references need attention:
-       Ref. 5: Author name should be "O JH" (not "O JH, et al." incorrectly split);
-       Ref.9: indicate supplement abstract and page/volume
-       Ref. 11: Please provide complete book chapter information (in: Computer Vision - ECCV 2014. Lecture Notes in Computer Science, vol. 8689. Springer; 2014:818-833)
-       Ref. 15 and others: please keep the correct diacritics (e.g., "Dührsen," "Müller").
-       Ref. 24 should clarify that it is an abstract/short communication (single page).
-       Ref. 26 must be corrected (proper citation is Biometrics. 1988;44:837-845).
-       Use full author initials consistently (e.g., "DeLong ER" not "Delong E").*

We thank the reviewer for pointing this out. We have made the corresponding changes in the references.
